# Application and research progress of single-port laparoscopy in retroperitoneal lymphadenectomy for gynecologic malignancies

**DOI:** 10.3389/fonc.2026.1824243

**Published:** 2026-05-11

**Authors:** Ruinan Song, Jiaqiang Xiong, Rourou Xiao, Yuexiong Yi, Jing Cheng, Xiwen Wang, Juyuan Huang, Jiahui Zhao, Qiongying Lyu, Yurou Chen, Wei Zhang

**Affiliations:** Department of Gynecologic Oncology, Zhongnan Hospital of Wuhan University, Wuhan, Hubei, China

**Keywords:** 3D laparoscopy, AI navigation, gynecologic neoplasms, retroperitoneal lymphadenectomy, robotic technology, single-port laparoscopy

## Abstract

Retroperitoneal lymphadenectomy has been widely used to eradicate locoregional disease, improve staging accuracy, and guide adjuvant therapy in the surgical treatment of gynecologic oncological patients. Although multi-port laparoscopy and open surgery can both effectively achieve retroperitoneal lymph node dissection, they are associated with greater surgical trauma and longer postoperative recovery, particularly in obese patients and those with prior surgical histories. In contrast, single-port laparoscopy is an innovative alternative surgical approach for retroperitoneal lymphadenectomy. This technique can offer potential advantages, such as improved cosmetic outcomes, reduced postoperative pain, faster recovery, and improvements in postoperative quality of life. Nonetheless, single-port laparoscopic retroperitoneal lymphadenectomy remains controversial. This article provides a narrative clinical review of the current evidence on single-port laparoscopy for retroperitoneal lymphadenectomy in gynecologic malignancies and is supplemented by a structured literature review and quantitative synthesis of selected comparative studies. Major concerns include the risk of injury to surrounding organs during difficult dissections, the completeness of lymph node removal, patient survival benefits, appropriate patient selection, and the learning curve. This review summarizes key anatomical considerations, current surgical techniques, and advancements in instrumentation, ongoing technical challenges, and future directions of single-port laparoscopic retroperitoneal lymphadenectomy.

## Introduction

Lymphatic dissemination is one of the primary metastatic pathways for gynecologic malignancies. Consequently, lymph node status directly influences disease staging, prognostic assessment, and postoperative treatment decisions (1). In the most recent updates, specifically the 2018 and 2023 revised staging criteria, the International Federation of Gynecology and Obstetrics (FIGO) has formally integrated lymph node status into the staging classification. These guidelines emphasize that accurate nodal assessment, whether achieved through high-quality imaging, sentinel lymph node (SLN) mapping, or systematic dissection, is critical for defining the extent of disease and guiding adjuvant radiochemotherapy (2, 3).Taking early-stage cervical cancer as an example, the five-year survival rate decreases from 88%–92% in node-negative patients to only 55%–64% in node-positive patients. The standard treatment for early-stage cervical cancer is radical hysterectomy combined with systematic pelvic lymphadenectomy. Pathological examination of surgical specimens determines the extent of metastasis, providing the basis for adjuvant radiochemotherapy (4).Similarly, systematic pelvic and para-aortic lymphadenectomy for endometrial and ovarian cancers is utilized not only for accurate staging but is also considered to improve disease-free survival in intermediate-to-high-risk patients. However, according to the LION trial published by Harter in 2019, in advanced ovarian cancer, systematic lymph node dissection does not improve survival and instead increases complications (5, 6).

Although traditional open or multi-port laparoscopic approaches can effectively perform lymphadenectomy, they require multiple abdominal incisions, resulting in greater trauma, slower postoperative recovery, and poorer cosmetic outcomes (7), Furthermore, the multi-port design may be less advantageous for obese patients or those with a history of abdominal surgery (8). With the evolution of minimally invasive surgery and the increasing demand from both surgeons and patients for “scarless” surgery and rapid recovery, single-port laparoscopy (SPL), also known as laparoendoscopic single-site surgery (LESS), has emerged as a promising minimally invasive approach. In recent years, SPL has been widely used for benign gynecologic diseases and has even been applied to malignant tumor surgeries, such as radical hysterectomy and pelvic lymphadenectomy (9). By inserting a multi-channel trocar through the natural umbilical scar, this technique allows the entire surgical procedure to be completed within a single small incision. It has the advantages of superior incision cosmesis, reduced postoperative pain, and fewer complications. It also minimizes bowel interference and lowers the incidence of intra-abdominal visceral injury and pelvic adhesions, particularly beneficial for obese patients. This improvement upon traditional laparoscopy not only achieves the goal of “hidden scars” but also confers a better postoperative quality of life for patients (7, 10). However, the transition of SPL from benign to malignant gynecologic surgery has been accompanied by unresolved controversies: while short-term perioperative benefits are well-documented, concerns persist regarding its ability to achieve radical lymphadenectomy in complex anatomical regions, such as deep obturator fossa, presacral area, supra-renal para-aortic region and the lack of high-quality evidence confirming long-term oncologic efficacy (11). Therefore, this article primarily provides a narrative clinical review of the anatomical basis, surgical techniques, clinical applications, controversies, and future directions of SPL for retroperitoneal lymphadenectomy in gynecologic malignancies. To complement this narrative synthesis with comparative evidence, we also performed a structured literature review and quantitative synthesis of 8 selected studies evaluating perioperative, lymph node, and oncologic outcomes.

## Literature search and quantitative evidence synthesis

To complement the narrative clinical review with comparative clinical evidence, we performed a structured literature review and quantitative synthesis of selected studies evaluating SPL for retroperitoneal lymphadenectomy in gynecologic malignancies.

### Literature search strategy

A structured literature search was conducted in PubMed, Embase, Web of Science, and the Cochrane Library from database inception to October 31, 2025. The search strategy combined controlled vocabulary and free-text terms related to single-port surgery, laparoendoscopic single-site surgery (LESS), retroperitoneal lymphadenectomy, pelvic lymphadenectomy, para-aortic lymphadenectomy, and gynecologic malignancies. Representative search terms included “single-port laparoscopy”, “single-site laparoscopy”, “laparoendoscopic single-site surgery”, “LESS”, “pelvic lymphadenectomy”, “para-aortic lymphadenectomy”, “retroperitoneal lymphadenectomy”, “endometrial cancer”, “cervical cancer”, and “ovarian cancer”. The search strategy was adapted as appropriate for each database.

### Eligibility criteria

Studies were considered eligible for the quantitative synthesis if they met the following criteria: 1) Population: Patients with histologically confirmed gynecologic malignancies (cervical, endometrial, or ovarian cancer) undergoing retroperitoneal lymphadenectomy. 2) Intervention: single-port laparoscopy retroperitoneal lymphadenectomy. 3) Comparison: Traditional multi-port laparoscopy lymphadenectomy. 4) Outcomes: At least one of the following outcomes was reported: perioperative parameters (operative time, estimated blood loss, length of hospital stay), oncologic outcomes (lymph node yield, recurrence, survival), complications, or patient-reported outcomes (cosmetic satisfaction). 5) Study Design: prospective or retrospective cohort studies, as well as randomized control trials (RCT). Studies were excluded if they were: (1) single-arm studies without a control group; (2) conference abstracts, case reports, or reviews; (3) studies with insufficient data for extraction; or (4) Studies utilizing robot-assisted laparoscopic platforms.

### Study selection and data extraction

Two authors (R.S. and R.X.) independently screened titles and abstracts, followed by full-text assessment of potentially eligible studies. Disagreements were resolved through discussion and, when necessary, consultation with a third reviewer. Data extracted from the included studies comprised author, publication year, study design, patient population, cancer type, sample size, surgical approach, extent of lymphadenectomy, and the outcomes of interest.

### Risk-of-bias assessment

The methodological quality of included observational studies was assessed using the Newcastle-Ottawa Scale (NOS), with scores of ≥7 indicating high quality. The risk of bias for RCT was evaluated using the modified Jadad scale. (**Supplementary Table S1**).

### Quantitative synthesis and statistical analysis

Quantitative synthesis was performed for outcomes reported by sufficiently comparable studies. For continuous variables, pooled mean differences (MDs) with 95% confidence intervals (CIs) were calculated. For dichotomous outcomes, pooled odds ratios (ORs) with 95% CIs were used. Statistical heterogeneity was assessed using the I² statistic. A random-effects model was applied when substantial heterogeneity was present; otherwise, a fixed-effects model was used. Because of the limited number of available studies for several outcomes and the clinical heterogeneity across study populations and surgical settings, the pooled estimates should be interpreted as exploratory and supportive of the narrative synthesis rather than definitive measures of comparative effectiveness.

A PRISMA-style study selection process was used for the quantitative synthesis. After removal of duplicates, titles and abstracts were screened, followed by full-text assessment of potentially eligible studies. The final meta-analysis included 8 studies that met the predefined eligibility criteria. (**Supplementary Figure S1**).

## Anatomical basis of retroperitoneal lymphadenectomy via single-port laparoscopy

### Definition of the retroperitoneal space

**Figure 1:**
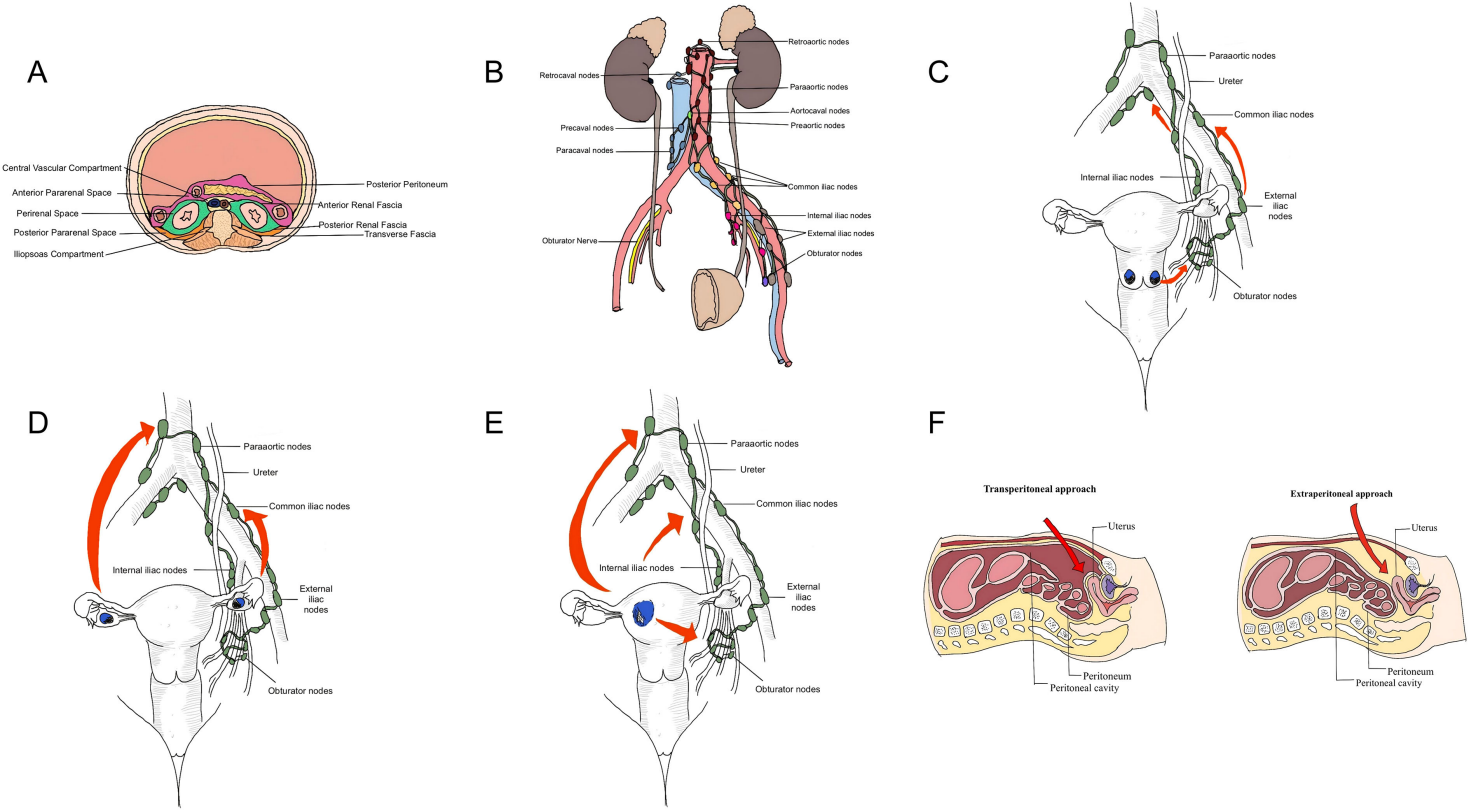
Anatomical basis, lymphatic spread patterns, and surgical approaches relevant to single-port retroperitoneal lymphadenectomy in gynecologic malignancies. **(A)** Schematic cross-sectional anatomy of the retroperitoneal space. **(B)** Distribution of major retroperitoneal lymph node groups. **(C)** Main lymphatic spread pattern in cervical cancer. **(D)** Main lymphatic spread pattern in ovarian cancer. **(E)** Main lymphatic spread pattern in endometrial cancer. **(F)** Schematic comparison of the transperitoneal and extraperitoneal approaches.

The retroperitoneal space is a three-dimensional area bounded anteriorly by the posterior peritoneum, posteriorly by the transversalis fascia, superiorly by the diaphragm, and inferiorly by the pelvic cavity. It contains the major vascular, neural, and lymphatic structures relevant to retroperitoneal lymphadenectomy, including the abdominal aorta, inferior vena cava, para-aortic and paracaval lymphatic chains, and iliac vascular regions (12). Retroperitoneal lymph nodes are located in the potential space posterior to the abdominal and pelvic cavities, serving as a transit station for lymphatic drainage. These lymph nodes are distributed primarily in the renal hilar region near the renal vessels and nerves, the para-aortic region arranged along both sides of the abdominal aorta, the paracaval region distributed around the inferior vena cava, and the peri-iliac vascular region located near the common iliac vessels at the pelvic inlet. Pelvic lymph nodes primarily course along the external iliac, internal iliac, and common iliac vessels, and cover the obturator nerve (13). These lymph node groups receive lymphatic drainage from the pelvic and abdominal organs as well as the abdominal wall, constituting the primary targets for dissection in gynecologic malignancies (**Figure 1A**). **Figure 1A** illustrates the major retroperitoneal fascial planes and compartments that define the anatomical working space for retroperitoneal lymphadenectomy.

### Relationship with key vessels and nerves

Pelvic lymph nodes are typically close to the vessels and cover the surface of the nerve (1). External Iliac Lymph Nodes: Arranged along the anteromedial and lateral regions of the external iliac vessels, extending from above the inguinal ligament to the bifurcation of the iliac artery. The medial group often adjoins the upper edge of the obturator nerve. Surgical dissection is typically performed close to the vascular sheath in a “lateral-to-medial, cranial-to-caudal” direction. (2) Internal Iliac Lymph Nodes: Distributed around the main trunk of the internal iliac artery and its branches. Surgeons must be careful when performing deep dissection of lymph nodes to prevent bleeding from the presacral venous plexus and traction injury to the nerve plexus. (3) Obturator Lymph Nodes: Located in the obturator fossa. This is one of the most common sites for pelvic metastasis in gynecologic oncology. (4) Common Iliac: Up along the common iliac vessels to the bifurcation of the aorta. They receive lymphatic output from both the external and internal iliac lymph nodes (14, 15).

Lumbar lymph nodes are divided into the left lumbar lymph nodes, intermediate lumbar lymph nodes, and right lumbar lymph nodes (**Figure 1B**). As shown in **Figure 1B**, these nodal groups are closely related to the abdominal aorta, inferior vena cava, ureters, and iliac vessels, which are key anatomical landmarks during retroperitoneal dissection. (1) The left lumbar lymph nodes are also known as para-aortic lymph nodes. Para-aortic lymph nodes are categorized into pre-aortic, retro-aortic, and lateral aortic nodes. The upper boundary of the preaortic lymph nodes is usually the left renal vein. Retro-aortic lymph nodes range from below the left renal vein to the aortic bifurcation. Lateral aortic nodes extend cranially to the left renal vein and caudally to the aortic bifurcation (16). (2) Intermediate lumbar nodes are positioned between the abdominal aorta and the inferior vena cava, usually located below the level of the renal vessels. (3) Right lumbar lymph nodes are also known as paracaval nodes. They are located lateral, anterior, and posterior of the inferior vena cava and receive lymphatic drainage from the right common iliac nodes, uterus, and ovaries.

The complex anatomical structures pose some challenge for SPL lymph node dissection (13). Proficiently identifying vascular pulsations, neural pathways, and lymph node anatomical landmarks is not only the foundation of surgical safety but also a prerequisite for addressing the controversy of dissection thoroughness.

## Metastasis patterns and dissection scope by tumor type

Lymphatic metastasis in cervical cancer initially spread to the obturator lymph nodes, followed by the internal and external iliac lymph nodes, and subsequently extended to the common iliac and para-aortic regions (**Figure 1C**). **Figure 1C** highlights the predominant involvement of the obturator and pelvic nodal basins in cervical cancer and illustrates the typical upward metastatic pathway. A retrospective study reported that the metastasis rate to obturator lymph nodes of patients with stage IB1 cervical cancer was as high as 30.9%, whereas the para-aortic metastasis rate was 0% (17). Another retrospective study found that the metastasis rate to the obturator region of patients with stage IB1 cervical cancer accounted for 42.5%, which was significantly higher than the internal iliac (20.3%) and external iliac regions (19.9%), while the common iliac (9.8%) and presacral (7.5%) nodes showed the lowest rates (18). These findings consistently suggest that the obturator region is the most frequent nodal basin involved in early-stage cervical cancer. In summary, emphasis in early-stage cervical cancer should be placed on pelvic lymphadenectomy (19). However, the lymphatic spread pattern changes in locally advanced cervical cancer (LACC). In these patients, the risk of distant lymphatic metastasis increases significantly, and reliance on imaging alone is insufficient due to a high rate of occult disease. A multicenter prospective study by Leblanc et al. demonstrated that among LACC patients with negative para-aortic nodes on PET-CT, 12% were confirmed to have histological occult metastasis upon surgical staging (20). Furthermore, a systematic review indicated that in patients with proven pelvic nodal metastasis, the incidence of concurrent occult para-aortic involvement rises to approximately 16-25%, often evading detection by conventional imaging (21). For patients with locally advanced disease and negative para-aortic nodes on imaging, dissection may be extended to the level of the (inferior mesenteric artery) IMA for staging purposes.

Lymphatic metastasis in ovarian cancer spreads along retroperitoneal pathways, extending from the pelvis to the para-aortic region (**Figure 1D**). As illustrated in **Figure 1D**, this cephalad spread pattern provides the anatomical basis for para-aortic assessment in selected ovarian cancer patients. In ovarian cancer patients undergoing systematic lymphadenectomy, the incidence of para-aortic lymph node metastasis above the renal veins is approximately 16%. Notably, these cases are invariably associated with metastasis in the lower para-aortic and pelvic regions (22). This implies that if high-level lymph node involvement is suspected, intraoperative dissection should be extended to the level of the renal veins. This metastatic pattern raises a critical controversy for SPL application: can SPL reliably achieve complete dissection of upper para-aortic lymph nodes (above the renal veins) given its technical limitations? A feasibility study reported the use of a transumbilical single-port laparoscopic extraperitoneal approach for simultaneous pelvic and para-aortic lymphadenectomy in 8 high-risk ovarian or endometrial cancer patients, with a median of 18.0 para-aortic lymph nodes and 26.5 pelvic region lymph nodes retrieved, a median operative time of 166.5 minutes and no conversions to laparotomy. Patients experienced rapid recovery with no observed complications (23). However, the small sample size and lack of long-term follow-up prevent definitive conclusions about its adequacy for ovarian cancer’s extensive metastatic potential.

The scope of lymphatic metastasis in endometrial cancer encompasses both pelvic and para-aortic lymph nodes. The overall lymph node metastasis rate was 10.8%, 6.6% for para-aortic nodes, 5.5% for obturator nodes, 4.6% for internal iliac nodes, and 3.8% for common iliac nodes (24). Notably, when pelvic lymph nodes are positive, the risk of para-aortic involvement increases substantially (>40%), indicating the significant importance of para-aortic lymphadenectomy (25). (**Figure 1E**). **Figure 1E** illustrates the dual pelvic and para-aortic lymphatic drainage pattern of endometrial cancer.

FIGO staging and prognostic assessment are directly influenced by lymph node status. In early-stage cervical cancer, the 5-year survival rate is approximately 92% in patients without pelvic lymph node metastasis, compared to 64% in those with metastasis (26). Clinical studies have shown that the prognosis for this subtype is more favorable compared to other Stage III categories, but significantly inferior to that of the node-negative population (27, 28). In endometrial cancer, the survival rate is worse in patients with para-aortic lymph node metastasis compared to those with isolated pelvic nodal metastasis (29). Therefore, systematic retroperitoneal lymphadenectomy not only facilitates accurate staging but also provides the basis for decision-making regarding adjuvant radiochemotherapy.

## Development of single-port laparoscopic technology

### Single-port laparoscopic approaches and platforms

In 1969, transumbilical single-incision tubal ligation was introduced. In 2010, the technical feasibility of this approach for pelvic and para-aortic lymphadenectomy (30). There are two main surgical approaches in retroperitoneal surgery for gynecologic malignancies. The first is the transperitoneal approach, which is the conventional approach (**Figure 1F**). As shown in **Figure 1F**, the transperitoneal approach enters through the peritoneal cavity and therefore provides a familiar operative view but remains more susceptible to bowel interference. A broad field of view and well-defined anatomical landmarks are its advantages. However, it is easily susceptible to bowel interference, particularly during high para-aortic lymphadenectomies (31).

The second is the extraperitoneal approach, which is established between the posterior rectus sheath and the peritoneum via the umbilical incision (**Figure 1F**). In contrast, **Figure 1F** also demonstrates that the extraperitoneal approach creates a working space outside the peritoneal cavity, which may facilitate more direct retroperitoneal exposure. This approach effectively circumvents interference from intra-abdominal organs, reduces the risks of postoperative ileus and adhesions, and provides more direct and distinct exposure to the surgical field (32, 33).

A key controversy in surgical approach selection centers on which technique best balances comprehensive dissection with the minimization of complications. A meta-analysis comparing transperitoneal and extraperitoneal SPL found that the extraperitoneal approach achieved a significantly higher yield of para-aortic lymph nodes (12.7 ± 4.1 vs. 8.3 ± 3.7, P < 0.01) (34). However, the extraperitoneal approach has a steeper learning curve and greater technical complexity, which may lead to higher complication rates among surgeons with limited experience (33). Thus, the choice of approach is not merely a technical preference but a balance between dissection efficacy and surgeon proficiency—highlighting the need for standardized training and patient selection criteria.

## Surgical techniques and strategies for optimizing surgical field exposure

In SPL surgery, “exposure” entails not merely revealing the lesion but creating the operative space. SPL utilizes the Trendelenburg position. This position leverages gravity to displace the small intestine, omentum, and transverse colon cephalad. This maneuver allows the natural retraction of intra-abdominal gravitate away from the pelvic and lower abdominal regions. SPL employs an angle of 30° to 40°, which is steeper than the 15°–20° used in conventional laparoscopy. Such an inclination is conducive to achieving adequate exposure of the regions within the limited visual field associated with single-port access (13). Percutaneous suture suspension is the most fundamental and widely cited method. This method involves the use of a straight needle or a suture-carrying needle to penetrate the abdominal wall directly for suspension. The surgeon can dynamically adjust the direction of traction extracorporeally. This technique effectively preserves the operative space under low-pressure pneumoperitoneum or even gasless conditions, thereby avoiding the compression of major vessels caused by high intra-abdominal pressure. It is particularly suitable for elderly cancer patients with cardiopulmonary insufficiency. There is also controversy regarding exposure techniques. The Trendelenburg position may cause hypotension, venous air embolism, or respiratory compromise, especially in obese or comorbid patients (35). Additionally, dynamic suspension techniques require additional abdominal wall punctures, which potentially undermines the “scarless” advantage of SPL and increases the risk of adhesion formation (36). Thus, the selection of the exposure technique must balance surgical efficacy with patient safety. The choice of individualized decision-making should be based on patient characteristics and the surgeon’s experience.

## Challenges and solutions in single-port laparoscopy

Despite the continuous maturation of surgical techniques, retroperitoneal lymph node dissection via single-port laparoscopy faces inherent technical bottlenecks. However, these challenges have simultaneously catalyzed the development of diverse solutions.

### Main challenges

#### Instrument collision and restricted manipulation

All instruments of SPL are introduced through a single channel and positioned in parallel, making them highly prone to mutual collision and interference—a phenomenon known as the “chopstick effect” (37). The “Chopstick Effect” restricts the degrees of freedom in manipulation and significantly increases the difficulty of fine motor tasks such as suturing and knot tying (37, 38).

#### Limited field of view and lack of depth perception

The two-dimensional image from a single viewing angle fails to provide stereoscopic spatial perception, making it particularly difficult to judge anatomical planes, especially when addressing deep structures such as the obturator fossa and the level of the renal vessels.

#### Steep learning curve

Compared to traditional multi-port laparoscopy, SPL surgery forces surgeons to operate in an environment devoid of instrument triangulation and depth perception, placing exponentially higher demands on spatial imagination and hand-eye coordination(**Table 1**). Cumulative Sum (CUSUM) analyses indicate that establishing basic competency in endometrial cancer SPL surgeries requires approximately 11 cases, and achieving proficiency in complex cases necessitates over 40 cases (38). For radical hysterectomy for cervical cancer, surgeons must complete at least 15 cases to overcome the high-risk learning plateau (39). In robotic-assisted surgery and lymph node dissection, systemic proficiency still necessitates approximately 24 cases (40). Notably, lymph node dissection represents a rate-limiting step in the learning curve, with bilateral SLN mapping requiring 30 to 48 cases for mastery (41, 42).

**Table 1:** Comparison of learning curve thresholds for different surgical approaches and procedures.

Surgical approach	Procedure	Cases required for proficiency (approx.)
TU-LESS (Transumbilical Single-Site)	Endometrial Cancer Staging	11 cases
Manual LESS (“Chopstick Technique”)	Radical Hysterectomy	15 cases
RSRH (Robotic Single-Site)	Radical Hysterectomy	24 cases
SLN Mapping	Sentinel Lymph Node Biopsy	30–48 cases

The steep learning curve is a major barrier limiting the widespread adoption of SPL. Unlike multi-port laparoscopy, where proficiency can be achieved with fewer cases, SPL requires a substantial number of cases to ensure safe and effective lymphadenectomy. This creates a situation in which centers need to perform enough cases to gain proficiency, but the initial high-risk phase before overcoming the learning curve may deter adoption. Furthermore, there is no consensus on how to define proficiency, with proposed indicators including operative time, lymph node yield, and complication rate, leading to variability in training standards and clinical outcomes (38). In practical terms, adoption of SPL for retroperitoneal lymphadenectomy should follow a stepwise training pathway. Surgeons should first gain proficiency in conventional multi-port laparoscopy and less complex single-port procedures before progressing to oncologic staging cases and, subsequently, to more technically demanding retroperitoneal lymphadenectomy. Early adoption should be guided by careful case selection, structured mentoring, and, where available, simulation-based training. Such a progressive training strategy may help improve surgical safety, reduce variability during the learning phase, and support more standardized implementation of SPL.

## Solutions and technical innovations

### Instrument innovation

Traditional energy devices like ultrasonic scalpels and bipolar coagulation instruments have rigid, straight-shaft designs with limited maneuverability. Multi-DOF flexible instruments address this limitation. The Vessel Sealer Extend^®^ for the Da Vinci system has ±90° articulating jaws and 360° rotation, sealing vessels ≤7 mm. A 2025 prospective study found that this device reduced blood loss and increased lymph node yield compared to conventional monopolar scissors in high-risk endometrial cancer (43). The ArtiSential^®^ articulating instrument mimics wrist movement. Compared to pre-bent instruments, it offers more intuitive control and better force transmission, reducing the “chopstick effect” (44). However, a key controversy is cost-effectiveness. While these instruments improve maneuverability, their high cost may not be justified by marginal clinical benefits, especially in resource-limited settings. By incorporating multi-DOF wrist joints, they extend surgical reach and offer superior hemostasis with minimal thermal injury. However, these devices remain experimental, lacking widespread clinical validation. Advances in material science and actuation technology may enable them to overcome current limitations and enhance the clinical value of single-port laparoscopy.

### Visual system upgrades

3D laparoscopy provides stereoscopic vision, improving spatial perception and identification of critical structures. A meta-analysis of 25 RCTs found that 3D laparoscopy reduced overall operative time by approximately 8% (45). In Total Laparoscopic Hysterectomy (TLH), 3D shortened surgery by 13.7 minutes versus 2D, with no difference in complications (46). However, its benefit in SPL remains uncertain. A double-blind RCT of 68 cases found no significant differences in operative time (84.5 ± 20.5 vs. 87.8 ± 24.4min, *P* = 0.452), blood loss, or complications (47). Most studies also show no difference in lymph node retrieval. However, Long-term oncological outcomes (disease-free survival/overall survival[DFS/OS]) have not been compared in large trials (48–50). Therefore, it is unclear whether 3D improves dissection thoroughness or survival.

### Robotic single-port platform

In 2008, the da Vinci SP system was introduced, which uses a single cannula with a flexible 3D HD camera and three articulated instruments (51). Compared to early multi-port systems, it offers better maneuverability, improved deep-field visualization, and a shorter learning curve (52). Retrospective studies in gynecologic oncology demonstrate that the approach was feasible and safe for endometrial and ovarian cancers, with similar lymph node yields and complication rates. One study found significantly shorter operative times in the SP group versus the Xi group, with no additional complications or risks during follow-up (53, 54). Another reported a median lymph node yield of 12 with SP versus 5 with Xi, without significant differences in metastasis (55). A small series of 7 ovarian cancer cases showed no intraoperative conversions or complications, with a median stay of 3 days, blood loss of 10 mL, and 7 dissected nodes (56). The application in gynecologic oncology in China is limited. No evidence shows superior long-term outcomes over traditional robotic or laparoscopic approaches, and challenges, including high costs and narrow indications, persist. The learning curve also remains a barrier.

## Clinical application and efficacy evaluation of single-port laparoscopy

A summary of the main clinical studies directly comparing single-port laparoscopy and multi-port laparoscopy in gynecologic malignancies is provided in **Table 2**. Overall, the currently available comparative evidence suggests that SPL is feasible in selected patients and is generally associated with perioperative and short-term oncologic outcomes comparable to those of MPL, although most studies are retrospective, include relatively small or selected populations, and have limited long-term follow-up.

**Table 2:** Key studies comparing single-port laparoscopy and multi-port laparoscopy in gynecologic malignancies.

Author (year)	Cancer type	Study design	Sample size	Main perioperative findings	Main oncologic findings
Kang et al. (2023) (57)	Early-stage endometrial cancer	RCT	107 (53 vs 54)	OT, EBL, LOS, and complications were similar; pelvic LN yield met non-inferiority.	Median follow-up 34 months; PFS and OS were similar.
Cai et al. (2016)	Early-stage endometrial cancer	Retro	36 (18 vs 18)	Node yield, OT, EBL, complications, stay, and cost were similar; cosmetic satisfaction favored TU-LESS.	No recurrence during follow-up.
Hudry et al. (2013)	Mixed gynecologic cancers	Retro	69 (33 vs 36)	SPL showed shorter OT and hospital stay; EBL similar; node count similar after multivariate analysis.	No survival analysis reported.
Escobar et al. (2012)	Presumed early-stage endometrial cancer	Retro	60 (30 vs 30)	OT, EBL, LOS, and complications were similar; pelvic node yield was higher in SPL; para-aortic nodes similar.	No long-term oncologic outcomes reported.
Park et al. (2014)	Early-stage endometrial cancer	Retro	111 (37 vs 74)	Node yield, OT, EBL, transfusion, LOS, and complications were similar; pain and analgesic use were lower with LESS.	1 recurrence, in MPL group, after median 17-month follow-up.
You et al. (2023) (58)	Endometrial cancer	Retro	156 (78 vs 78)	Conversion, OT, EBL, nodes, and complications were similar; SPL had earlier ambulation, shorter catheter duration, less pain, and better incision satisfaction.	4-year DFS and OS were similar.
Cai et al. (2021) (17)	Type I stage I endometrial cancer	RCT	93 (31 vs 62)	SPL had longer OT but better postoperative recovery, less pain, and better cosmetic/QoL outcomes; node counts and complications were similar.	No OS or DFS difference; surgery type was not associated with prognosis in Cox models.

EC, endometrial cancer; EBL, estimated blood loss; LN, lymph node; LOS, length of stay; MPL, multi-port laparoscopy; OT, operative time; PFS, progression-free survival; OS, overall survival; PSM, propensity score matching; RCT, randomized controlled trial; SPL, single-port laparoscopy.

Based on these comparative studies, the perioperative, technical, and oncologic implications of SPL are discussed below. (**Table 2**).

### Perioperative outcomes

Single-port laparoscopy showed potential minimally invasive advantages in perioperative outcomes. Compared to multi-port laparoscopy, it significantly reduced postoperative length of stay (MD = -0.51 days, 95% CI: -0.90 to -0.12, P = 0.01), lowering medical costs and improving clinical turnover. A favorable trend was observed in reducing intraoperative blood loss, though not statistically significant(*P* = 0.09). Notably, despite the belief that single-port surgery takes longer, total operative time did not differ significantly between groups, likely due to improved surgical techniques and accumulated experience (**Table 3**).

**Table 3:** The meta-analysis of perioperative outcomes.

Category	Outcome measure	No. of studies	Pooled effect estimate [95% CI]	P-value	Conclusion
Perioperative Outcomes	Operative Time (min)	8	MD:10.39 [-19.52, 40.30]	0.5	No significant difference
	Estimated Blood Loss (mL)	8	MD: -11.52 [-24.72, 1.69]	0.09	Favors SPL (less blood loss), but not statistically significant
	Length of Hospital Stay (d)	8	MD: -0.51 [-0.90, -0.12]	0.01	Significantly shorter stay for SPL

However, these perioperative findings should be interpreted with caution. Most of the available studies were retrospective and were conducted in selected patient populations, which may have introduced selection bias. In addition, operative time and perioperative performance are strongly influenced by surgeon experience, institutional case volume, and stage within the learning curve (59). Although a shorter hospital stay may reduce some direct healthcare costs, this potential benefit may be offset by the higher cost of specialized SPL instruments and platforms while specialize (60). Therefore, perioperative outcomes should not drive SPL adoption alone, long-term oncologic outcomes and cost-effectiveness must also be considered.

### Lymph node dissection

Single-port laparoscopy showed a slightly higher total lymph node yield than multi-port group (*MD* = 1.92, *95%CI:* 0.01 to 3.84, *P* = 0.05). A comparative study showed that the single-port retroperitoneal approach yielded significantly more para-aortic lymph nodes than the transperitoneal multi-port approach (12.7 ± 4.1 vs. 8.3 ± 3.7, *P* < 0.01). In contrast, lower para-aortic lymph node clearance was observed in single-port versus multi-port surgery (*MD* = -0.96, *P* = 0.002). Together, these findings suggest that SPL can achieve lymph node yields broadly comparable to those of conventional approaches in selected settings, although the results remain somewhat heterogeneous (**Table 4**). However, whether lymph node yield can be regarded as an adequate surrogate marker of oncologic adequacy remains uncertain. Although SPL achieves comparable or slightly higher yields in some studies, yield alone does not ensure removal of all metastatic nodes, including micrometastases (61). In addition, lymph node counts may be influenced by multiple factors, including the extent of dissection, tumor type, patient anatomy, surgical approach, surgeon experience, and pathological processing. Therefore, lymph node yield should be interpreted as a useful technical indicator rather than definitive evidence of oncologic equivalence. For example, a study using the “Renal Vein Angle” standardized SPL technique reported a median para-aortic lymph node yield of 21, but with only 15 months of median follow-up and no recurrence data (62). These limitations indicate that further validation using long-term oncologic endpoints is still needed.

**Table 4:** The meta-analysis of lymph node dissection.

Category	Outcome measure	No. of studies	Pooled effect estimate [95% CI]	P-value	Conclusion
Lymph Node Dissection	Pelvic Lymph Nodes Retrieved	8	MD: -0.98 [-2.33, 0.37]	0.16	No significant difference
	Para-aortic Lymph Nodes Retrieved	6	MD: 0.93 [-0.64, 2.50]	0.24	No significant difference
	Total Lymph Nodes Retrieved	3	MD: 1.92 [0.01, 3.84]	0.05	Significantly more nodes retrieved by SPL

### Oncological prognosis

Existing evidence validates the reliability of SPL according to surgical safety and long-term oncological outcomes. The results clearly indicate that there are no statistically significant differences between the two modalities in terms of overall complication rate, conversion rate to laparotomy, and tumor recurrence rate (Progression-Free Survival [PFS]/DFS), and long-term mortality rate (OS). The analysis of these core safety and efficacy indicators showed extremely low heterogeneity *(I²* = 0%). The low statistical heterogeneity observed in some pooled analyses may support the internal consistency of these findings. However, this should be interpreted cautiously in view of the limited number of studies and the clinical similarity of the included populations. High-quality evidence from RCTs shows that, in early-stage endometrial cancer, the 3-year PFS was 96.2% for SPL versus 98.1% for conventional MPL (P = 0.55), and the 3-year OS was 98.1% versus 100.0% (P = 0.31); neither difference was statistically significant (57). This finding is further supported by large-sample Propensity Score Matching (PSM) studies. Among 881 patients with endometrial cancer, the 3-year and 5-year DFS and OS in the single-port group showed no significant differences compared to multi-port, robotic, and open surgery groups after matching, with similar recurrence rates (63). Another matched-pairs study yielded consistent results (58). Recent retrospective studies and systematic reviews indicate that single-port surgery is non-inferior to laparotomy in terms of long-term survival outcomes for patients with early-stage ovarian cancer who have undergone adequate evaluation and completed standard staging (64). On the robotic platform, retrospective long-term follow-up comparisons between robotic single-port and multi-port laparoscopy also revealed no differences in DFS or OS, indicating that in experienced research centers, the single-port approach possesses a long-term safety profile equivalent to that of traditional minimally invasive pathways (65). Studies from the past five years universally demonstrate that, provided standard staging (including pelvic/para-aortic lymph node dissection or sampling) is completed, single-port laparoscopy is non-inferior to traditional multi-port laparoscopy or robotic surgery in terms of long-term oncological outcomes, and in most cases, is also not inferior to open surgery (**Table 5**).

**Table 5:** The meta-analysis of complications and long-term oncologic outcomes.

Category	Outcome measure	No. of studies	Pooled effect estimate [95% CI]	P-value	Conclusion
Complications	Overall Complication Rate	6	OR: 0.94 [0.50, 1.76]	0.84	No significant difference in risk of complications
	Conversion to Laparotomy Rate	3	OR: 1.28 [0.33, 4.87]	0.72	No significant difference in risk of conversion
Long-term Oncologic Outcomes	Recurrence Rate	5	OR: 1.28 [0.71, 2.33]	0.41	No significant difference in risk of recurrence
	Mortality Rate	4	OR: 0.73 [0.27, 1.97]	0.54	No significant difference in risk of mortality

Despite these reassuring findings, critical controversies and evidence gaps remain (1): Most studies have short follow-up durations (median 34–45 months), which may be insufficient to detect differences in recurrence for gynecologic malignancies with long natural histories (e.g., ovarian cancer) (58). (2) Most available studies were retrospective, involved relatively small or highly selected cohorts, and predominantly included early-stage or low-risk patients (63). (3) The lack of RCTs with long-term follow-up (≥5 years) means that non-inferiority of SPL to MPL or open surgery cannot be definitively confirmed. (4) There is no data on whether SPL influences the risk of isolated lymph node recurrence, which is a key endpoint for evaluating dissection thoroughness (11). Thus, while current evidence supports the safety of SPL in selected patients, definitive confirmation of its oncologic equivalence requires longer follow-up and inclusion of high-risk populations.

### Patient satisfaction

The most intuitive advantage of SPL is its superior aesthetic outcomes. By performing the surgery through a single small incision at the umbilicus, “scar concealment” is achieved, which greatly satisfies patients’ demands for postoperative cosmesis and significantly improves patient satisfaction. The meta-analysis results indicate that the satisfaction scores regarding incision cosmesis in the single-port group were significantly higher than those in the multi-port group (*SMD* = 2.62; *95% CI*: 0.68 to 4.55, *P* = 0.008) (**Table 6**). This substantial effect size indicates that the “scarless” appearance associated with single-port surgery effectively addresses body image concerns among gynecologic oncology patients. Simultaneously, reduced surgical trauma contributes to decreased postoperative pain. A comparative study recorded a mean Visual Analog Scale (VAS) score of 3.24 in the multi-port group, whereas it was only 1.32 in the single-port group. This generally implies milder pain perception by patients and a correspondingly reduced requirement for analgesics. The mitigation of postoperative discomfort directly supports the principles of Enhanced Recovery After Surgery (ERAS).

**Table 6:** The meta-analysis of cosmetic satisfaction score.

Outcome measure	No. of studies	Pooled effect estimate [95% CI]	P-value	Conclusion
Cosmetic Satisfaction Score	3	SMD: 2.62 [0.68, 4.55]	0.008	Significantly higher satisfaction in SPL

Patient satisfaction with SPL raises questions about whether aesthetic benefits justify potential oncologic risks. While patient-reported outcomes such as cosmesis and pain are important, they must be balanced against the primary goal of cancer treatment—curative intent. For example, a patient may prioritize a scarless outcome, but if SPL is associated with a higher risk of understaging (even if unproven), this choice may compromise long-term survival. Therefore, shared decision-making is critical, with full disclosure of both the benefits and uncertainties of SPL. Additionally, the lack of standardized patient-reported outcomes assessment tools for SPL in gynecologic oncology limits cross-study comparisons of satisfaction (60).

In summary, when applied to retroperitoneal lymph node dissection for gynecologic malignancies, SPL not only provides oncological radicality and surgical safety comparable to traditional MPL, but also confers significant clinical benefits to patients, including faster postoperative recovery and shorter hospital stays. However, critical controversies regarding the thoroughness of dissection in high-risk disease and cost-effectiveness, as well as evidence gaps related to long-term RCT data and patient-reported outcomes standardization, must be addressed before widespread adoption.

## Extensions and applications

### Lymph node dissection guided by specific tumor scenarios and molecular classification

In recent years, personalized treatment for gynecologic malignancies have increasingly focused on the importance of genetic and molecular profiles. Molecular classification not only predicts prognosis and recurrence risk but also the incidence of lymph node metastasis and the extent of dissection required. A 2024 study of 120 endometrial cancer patients found that the p53-abnormal subtype was significantly associated with the risk of lymph node metastasis. Multivariate analysis showed that p53-wild type serves as a protective factor against lymph node metastasis, whereas p53 mutations significantly increase the risk of metastasis. Systematic reviews further confirm that the p53-abnormal group has the highest rate of lymph node involvement, while the POLE-mutant group exhibits the lowest. Consequently, patients with p53 abnormalities and deep myometrial invasion should undergo full lymph node staging and postoperative adjuvant therapy (66). Given the low metastasis risk in POLE-mutant and p53-wild type tumors—and their excellent prognosis—some studies suggest chemotherapy may be unnecessary even if metastasis is found, and in select cases, SLN mapping or lymph node dissection could be omitted (67).

Molecular classification adds a new dimension to the controversy of SPL application: whether it is safe for high-risk molecularly subtypes like p53-abnormal endometrial cancer, which require a comprehensive lymphadenectomy. Currently, no evidence shows that SPL achieves dissection thoroughness in these groups compared to MPL or open surgery (63). However, molecular profile also provides an opportunity to personalize SPL use: From a future-oriented perspective, molecular classification may help refine surgical decision-making for retroperitoneal staging. Patients with lower-risk molecular profiles, such as POLE-mutant tumors, may represent particularly suitable candidates for SPL or even less extensive nodal assessment strategies. In contrast, for high-risk subtypes such as p53-abnormal tumors, in which comprehensive staging and oncologic thoroughness are paramount, conventional multi-port or robotic approaches may remain preferable until stronger SPL-specific evidence becomes available. This represents a key direction to resolve SPL controversies: tailoring surgical approach to molecular subtype, thereby optimizing patient selection and addressing concerns about dissection thoroughness in high-risk disease. However, a major evidence gap remains: no published studies have evaluated perioperative or oncologic outcomes of SPL stratified by molecular subtype. This gap is clinically important, because molecular classification may help identify patients in whom the minimally invasive benefits of SPL can be maximized without compromising oncologic adequacy. Future prospective studies should therefore incorporate molecular subtype as a predefined stratification factor when evaluating patient selection, extent of nodal assessment, and long-term outcomes after SPL.

### Integration with other technologies

#### Radiomics sub-regional mapping

Traditional imaging relies on lymph node size criteria to diagnose metastasis, which yields limited sensitivity. In recent years, radiomics models based on MRI/CT and integrated with machine learning have emerged analyzing microscopic features, such as grayscale patterns and textures, within the tumor and peritumoral tissues to predict lymph node metastasis (LNM). Large-scale studies based on regional distribution indicate that in FIGO stage IB1 cervical cancer, the obturator group is the most frequent site of metastasis(42.5%), with a patient-level metastasis rate of 8.5%, significantly higher than the external iliac group (4.7%) and internal iliac group (5.4%) (18). In other words, the risk in the obturator group is approximately 1.8 times that of the external iliac group. This may provide an anatomical basis for more individualized nodal assessment and targeted surgical planning in SPL. On this basis, radiomics demonstrates stable performance in the preoperative prediction of “high-risk nodal groups in the lower pelvis centered on the obturator region.” A multi-center MRI fusion model achieved Area Under the Curve (AUC) values of 0.891, 0.863, and 0.804 for the training, internal validation, and external validation cohorts, respectively (68). This suggests robust discriminatory capability for high-risk obturator-internal iliac-external iliac nodal groups. A 2025 MRI radiomics study on cervical cancer reported that a training AUC of approximately 0.80 and a validation AUC of approximately 0.81 (69), while a 2024 retrospective study achieved an AUC of 0.923 in the training set and 0.820 in the test set (70). A 2021 CT radiomics study reported sensitivities of 0.854 and 0.870, and AUCs of 0.912 and 0.859 in the training and internal validation cohorts, respectively (71). However, most radiomics studies remain retrospective and model-based, and their direct clinical application to intraoperative decision-making in SPL retroperitoneal lymphadenectomy has not yet been established.

#### Intraoperative navigation systems

Fluorescence Tracers: SLN mapping using Indocyanine Green (ICG) enables real-time visualization of lymphatic drainage pathways, significantly improving the accuracy of identification and sampling for minute lymph nodes. In gynecologic oncology surgeries, the detection rate of ICG-guided lymph nodes reaches 95%–100%, with a false-negative rate of only 0%–8% and a negative predictive value as high as 97%–100%, demonstrating superiority over dissection based solely on anatomical experience (72). Furthermore, the detection rate of SLNs using ICG fluorescence imaging is 10% higher than that of traditional white-light visualization (73). This capability allows for precise lymph node localization even within the confined field of view typical of single-port laparoscopy, thereby reducing omissions, enhancing the thoroughness of dissection, and lowering the risk of postoperative recurrence.

Radioactive Tracers: Traditional radionuclides (e.g. ^99m^Tc) combined with a gamma probe enable preoperative imaging of lymphatic drainage and precise intraoperative detection of radiation signals. A study utilizing a hybrid ICG-^99m^Tc probe detected 97.1% of SLNs via the radioactive component and 80% via the fluorescence component; the complementarity of the two significantly improved the overall detection rate (74). In clinical practice, ^99m^Tc is frequently combined with blue dye or ICG to leverage the dual advantages of visual indication and deep-tissue detection.

Magnetic Tracers: Superparamagnetic Iron Oxide (SPIO) nanoparticles used with a handheld magnetometer, allow quantitative detection of magnetic signals and present lymph nodes with a brownish-black coloration. An early-stage cervical cancer study reported a 100% SLN detection rate (90% bilateral detection) with SPIO tracing, with sensitivity and AUC for metastasis both reaching 100% (75). No significant associated adverse events were observed, indicating that SPIO technology possesses excellent potential for SLN imaging in gynecologic cancers (4). Molecular Targeted: Fluorescence probes targeting tumor-specific receptors, such as pafolacianine (OTL38) targeting folate receptor alpha (FRα) improve the detection of microscopic lesions, with approximately 33% of patients having additional cancerous lesions identified during surgery (76).

Intraoperative navigation systems address the SPL controversy of limited visual field by enhancing the visibility of lymph nodes and critical structures. However, controversies persist: The added cost of tracers and equipment may not be feasible in all centers. There is no consensus on the optimal tracer for different gynecologic malignancies (e.g., ICG vs. SPIO for cervical cancer). The learning curve for using these systems in SPL (especially combining navigation with instrument manipulation) may be steep, potentially increasing operative time initially. Additionally, an evidence gap exists regarding whether navigation-guided SPL reduces recurrence rates compared to conventional SPL or MPL.

#### AI-assisted real-time navigation

With the advancement of AI technology, intraoperative real-time navigation is increasingly being utilized to enhance the safety and precision of single-port surgery. By registering preoperative images (SPECT/CT, CT, or MRI) with intraoperative visuals and superimposing them via Augmented Reality (AR), surgeons can visually localize suspected lymph nodes in a three-dimensional anatomical context. This may facilitate more targeted sampling or dissection in selected settings, rather than relying solely on broad anatomical clearance (77). Deep learning-based image recognition systems can real-time identify retroperitoneal anatomical structures, providing color-coded overlays. A 2023 prospective study found that AI navigation reduced the failure rate of the right hypogastric nerve by trainees from 37.5% to nearly 0% (78). AI can also integrate preoperative imaging with patient physiological data to assess bleeding risks and predict complications (79). AI-assisted navigation is a promising investigational tool that may help address some technical limitations of SPL, such as restricted field of view and intraoperative anatomical orientation. However, its value in improving dissection thoroughness or oncologic outcomes has not yet been established. However, AI systems remain in the early exploratory stage and lack large-scale clinical validation for SPL lymphadenectomy. Ethical and legal concerns exist regarding algorithm bias and error liability.

## Limitations, controversies, and development

At present, SPL appears most suitable for carefully selected patients treated in experienced centers, whereas its role in high-risk disease, extensive upper para-aortic dissection, and broader routine adoption remains uncertain.

### Thoroughness of lymph node dissection

The primary controversy surrounding SPL retroperitoneal lymphadenectomy is whether it achieves the same level of dissection thoroughness as traditional approaches, particularly in complex anatomical regions (deep obturator fossa, presacral area, supra-renal para-aortic region). While small-sample studies report comparable lymph node yields, concerns persist about missed micrometastases due to limited visualization and instrument maneuverability (23, 62). In addition, lymph node yield itself may not fully reflect oncologic adequacy, especially when the extent of dissection and the anatomical difficulty of the target region vary across studies. Other scholars compared lymph node yields and complications between SPL and MPL for radical hysterectomy and found no significant differences. However, the cases studied were predominantly early-stage cervical cancers and mostly did not involve presacral or high para-aortic lymph node dissection (39). Therefore, current evidence remains insufficient to confirm equivalent dissection adequacy in more technically demanding or higher-risk settings.

### Patient selection for SPL

There is no consensus on which gynecologic cancer patients are optimal candidates for SPL. Key debates include: (1) Whether SPL is appropriate for high-risk subtypes (e.g. p53-abnormal endometrial cancer, serous ovarian cancer) that require comprehensive lymphadenectomy. (2) The role of SPL in obese patients or those with prior abdominal surgery—while SPL may reduce adhesion risk, technical challenges may compromise dissection thoroughness. (3) Whether early-stage cervical/ovarian cancer patients with high-risk features (e.g., lymphovascular space invasion) should be excluded from SPL. Evidence gaps: (1) No guidelines or algorithms for patient selection based on tumor characteristics, patient factors, and surgeon experience. (2) Lack of studies stratifying outcomes by patient risk profile.

### Lack of standardized surgical protocols and comprehensive training system

Given the distinct differences in manipulation patterns, field exposure, and surgical strategies between SPL and traditional laparoscopy, unified standardized protocols and training systems are currently lacking. Learning curve analysis from Southwest Hospital indicates that establishing basic technical competency for trans-umbilical SPL in endometrial cancer requires approximately 11 cases, whereas the learning curve for radical hysterectomy using the “chopstick technique” reaches a steady state after the 15th case (38, 39). Due to the high technical difficulty of SPL, it should be adopted in a stepwise manner by teams possessing extensive laparoscopic experience. Novices should accumulate experience starting with simpler procedures, such as hysterectomy, before progressing to complex operations like lymph node dissection (38). Currently, there is a clinical absence of technical standards, tiered training, and credentialing specifically for SPL retroperitoneal lymph node dissection. This results in significant heterogeneity in surgeon experience and variability in postoperative complication rates and dissection quality. To promote this technology, Standardized Operating Procedures (SOPs) covering preoperative assessment, instrument configuration, key operative maneuvers, and complication management should be formulated. Furthermore, a multi-center training and quality monitoring system should be established, integrating simulation training and evidence-based learning curve evaluations. Additionally, the future introduction of emerging technologies, such as robotic single-port platforms, AI-assisted navigation, and flexible energy devices, will necessitate corresponding training programs and guidelines to ensure the consistency of surgical safety and efficacy.

## Conclusions

In summary, SPL for retroperitoneal lymphadenectomy in gynecologic oncology offers important minimally invasive advantages, including improved cosmesis, reduced postoperative pain, and faster recovery, while current evidence suggests generally comparable short-term oncologic outcomes in selected patients. However, unresolved concerns remain regarding dissection thoroughness in technically demanding regions, its role in high-risk disease, and the lack of robust long-term comparative data.

Future progress will likely depend not only on technological advances such as robotic platforms, 3D visualization, and AI-assisted navigation, but also on more precise patient selection strategies. In particular, determining whether molecular classification can help guide the choice of SPL represents a key future research priority. Such an approach may help identify lower-risk patients who are most likely to benefit from SPL while reserving more conventional approaches for biologically aggressive subtypes, such as p53-abnormal tumors, until stronger evidence is available. This may represent an important step toward more individualized and oncologically sound surgical management in gynecologic oncology.
