# Supplementary material for: Application and research progress of single-port laparoscopy in retroperitoneal lymphadenectomy for gynecologic malignancies
Source: Front Oncol. 2026 May 11;16:1824243. doi: 10.3389/fonc.2026.1824243 (PMC13199314; doi:10.3389/fonc.2026.1824243)
Supplement: Supplementary file 1 [file Table1.docx]

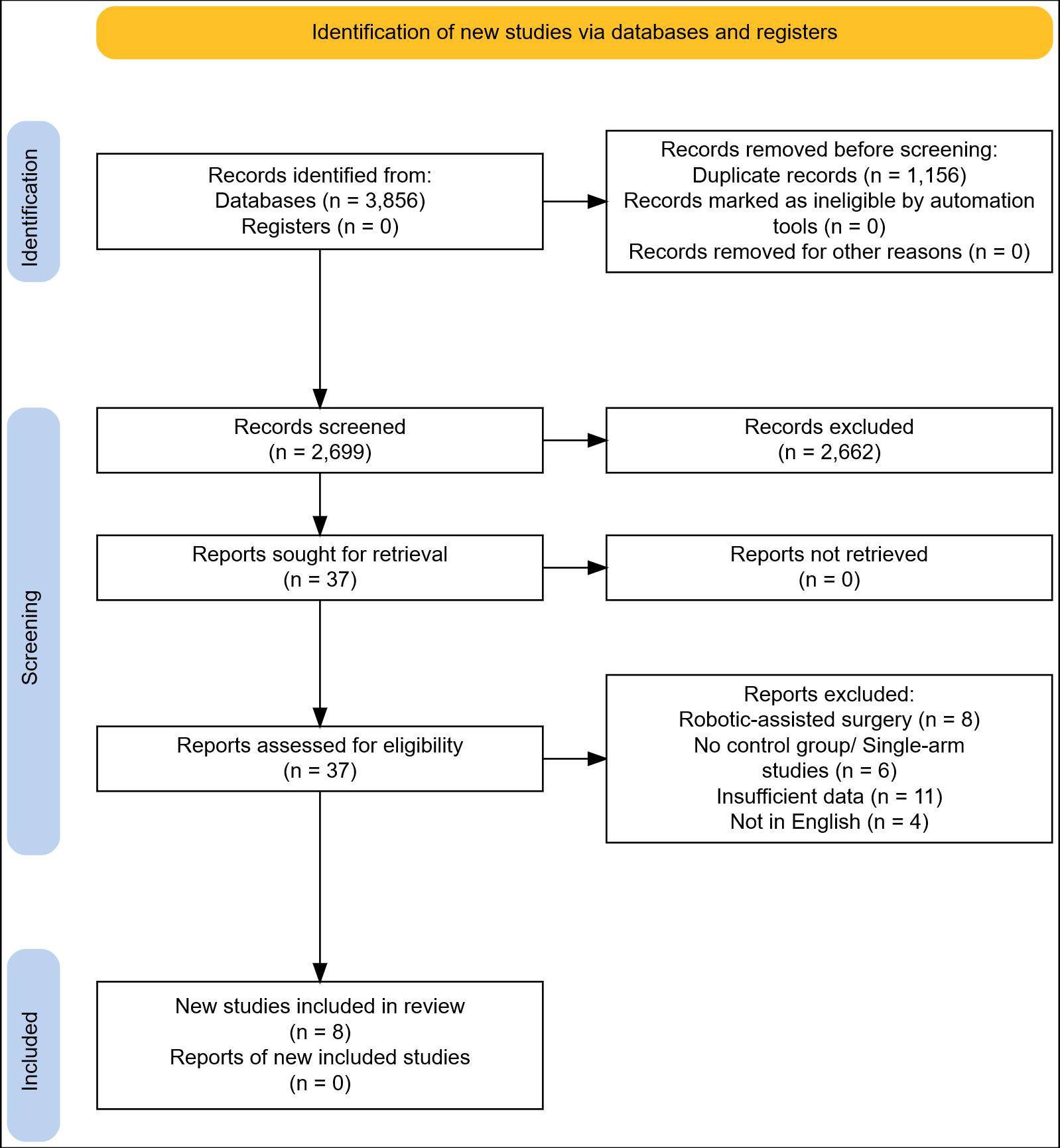


Supplementary Figure S1. PRISMA 2020 flow diagram of the literature search and study selection process.

Supplementary Table S1. Quality assessment of the included studies.

| **Study** | **Year** | **Design** | **Selection** | **Comparability** | **Outcome** | **Total score** | **Quality** |  |
| --- | --- | --- | --- | --- | --- | --- | --- | --- |
| **Randomized Controlled Trials (RCTs)** |  |  |  |  |  |  |  |  |
| Kang et al. | 2023 | RCT | 2 | 2 | 4 | 8 | High |  |
| Cai et al. | 2021 | RCT | 2 | 0 | 4 | 6 | Moderate |  |
| **Observational Studies (NOS)** |  |  |  |  |  |  |  |  |
| Hudry et al. | 2013 | Retro | **** | * | ** | 7 | High |  |
| Cho et al. | 2023 | Retro | **** | * | ** | 7 | High |  |
| Escobar et al. | 2012 | Retro | **** | * | *** | 8 | High |  |
| You et al. | 2023 | Retro | **** | * | ** | 7 | High |  |
| Park et al. | 2013 | Retro | **** | ** | *** | 9 | High |  |
| Cai et al. | 2016 | Retro | **** | ** | *** | 9 | High |  |
| RCT: Randomized controlled trial;Retro: Retrospective cohort study;  Note: RCTs were assessed using the modified Jadad scale (Range 0-8; Score ≥ 4 indicates high quality). Cai et al. did not explicitly describe the blinding method, resulting in a lower score in that domain compared to Kang et al. Observational studies were assessed using the Newcastle-Ottawa Scale (NOS) (Range 0-9; Score ≥ 7 indicates high quality). | | | | | | | |  |
|  |  |  |  |  |  |  |  |  |
|  |  |  |  |  |  |  |  |  |
|  |  |  |  |  |  |  |  |  |
|  |  |  |  |  |  |  |  |  |
